# An 18-Month Prospective Evaluation of a Novel Hyaluronic Acid Filler (YYS 720) for 3-Dimensional Nasal and Chin Augmentation

**DOI:** 10.1093/asjof/ojag146

**Published:** 2026-07-14

**Authors:** Minjeong Son

## Abstract

Hyaluronic acid (HA) fillers typically require frequent reinjections. Next-generation fillers, like the Y-phasic YVOIRE^®^ Y-solution (YYS) 720, require extended clinical validation to confirm their long-term performance and safety. This study evaluated 18-month clinical outcomes, safety, and satisfaction of YYS 720 for nasal and chin augmentation. In this prospective, single-arm study, 16 Korean patients received YYS 720 for the nose (*n* = 12) and chin (*n* = 7). Efficacy was evaluated through practitioner-assessed Global Aesthetic Improvement Scale (GAIS), patient-reported FACE-Q™ questionnaires, and objective 3-dimensional morphometric analysis. Safety was monitored through adverse event reporting and visual analog scale (VAS) pain scores. The GAIS responder rate was 100% through Month 12 and 84.21% at Month 18. FACE-Q scores significantly increased until Month 18 for the nose (80.67 ± 24.35, *P* = .0015) and Month 6 for the chin (86.00 ± 18.43, *P* = .0156). Three-dimensional analysis showed significant long-term improvements, with 1.73 ± 1.11 mL chin volume at Month 18 (*P* = .0061) and a 3.99 ± 2.81° nasofrontal angle increase at Month 12 (*P* = .0015) compared with baseline. The mean VAS score was low (1.69 ± 1.40). Only transient swelling and tenderness occurred in 4 patients, with no long-term delayed or serious adverse events. This pilot study provides preliminary evidence supporting the 18-month efficacy and safety of YYS 720. Given its pilot nature, these findings do not constitute claims of long-term reliability or durability, which were not formally assessed.

Level of Evidence: 4 (Therapeutic)

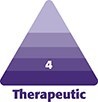

Hyaluronic acid (HA) fillers—the second most frequently performed nonsurgical aesthetic procedure—are the leading choice for facial contouring because of their biocompatibility and reversible nature.^1,2^ However, traditional HA fillers typically last only 6 to 12 months, necessitating frequent repeat injections.^3^ To address this, advanced crosslinking technologies have developed next-generation fillers with enhanced longevity, reducing patient discomfort and procedural frequency. Although extended persistence—confirmed by recent MRI-based studies—improves aesthetic satisfaction, it also demands rigorous long-term safety monitoring for delayed-onset adverse events (AEs).^4-6^ Consequently, clinical evaluation must now extend beyond short-term correction to verify sustained efficacy and safety.

The YVOIRE^®^ Y-solution (YYS) series was developed to meet this need. Featuring high elastic modulus (*G*′) and tissue integration, YYS 720 is a suitable candidate for areas requiring precise shape retention against dynamic forces, such as the nose and chin.^7^ Notably, the YYS series has demonstrated a favorable safety profile, with a reported delayed-onset AE rate of 0.1%.^8^

Despite the increasing longevity of modern fillers, many clinical trials still rely on short-term data. The present study addresses this gap by providing the first long-term, prospective evaluation of YYS 720 for nose and chin augmentation over an 18-month period to evaluate its clinical performance.

## METHODS

### Study Design and Participants

This prospective, single-arm, within-subject study evaluated the 18-month longitudinal performance of YYS 720 (LG Chem, Ltd, Seoul, Republic of Korea). Conducted at a single clinic in Changwon (Republic of Korea) between November 2023 and July 2025, the study enrolled 16 Korean adults (ages 20-70) seeking nose or chin augmentation. Participants received the filler treatment at no cost and small compensation for participation at each follow-up visit. Key exclusion criteria included previous filler treatment within 5 years or hypersensitivity to HA or lidocaine. Following baseline (V0), evaluations occurred immediately postprocedure (V1), at 2 to 4 weeks (V2; optional retouching), and at 3, 6, 12, and 18 months (V3-V6).

The study adhered to the Declaration of Helsinki and the International Council for Harmonisation—Good Clinical Practice guidelines, and all patients provided written informed consent.

### Treatment

Patients received YYS 720 (20 mg/mL of crosslinked HA and 3 mg/mL of lidocaine) injections administered by a board-certified aesthetic clinician into the nose and/or chin. For nasal augmentation, a 23G blunt-tip cannula targeted the deep fat layer (Layer 4) through nasal tip and glabellar entry points to create a uniform dorsal line. The nasal tip was projected through columellar injection to increase the nasolabial angle, with optional superficial placement to soften lateral contours. Entry points at the nasal tip and glabella were planned to achieve accurate shaping and structural definition.

For chin contouring, a dual-plane approach was used to achieve structural support and refined surface blending. A 23G cannula delivered YYS 720 to the premental space and along the periosteal plane to redefine the jawline. For midline projection, a 27G sharp needle provided deep bolus injections into the supraperiosteal layer of the mentum.

The injection volume was customized per patient. Retouching was performed as needed for desired correction. All procedural details, including date, volume, and technique, were documented.

### Efficacy and Safety Assessments

The primary endpoint was the Global Aesthetic Improvement Scale (GAIS) responder rate at 18 months (V6). Secondary endpoints included GAIS rate from V1 to V5, 3-dimensional (3D) morphometric analysis, and FACE-Q satisfaction scores.

#### Global Aesthetic Improvement Scale (GAIS)

Treatment outcomes were evaluated on a 5-point GAIS: 1 (worsened), 2 (unaltered), 3 (improved), 4 (very improved), and 5 (exceptional improvement) by practitioner. To provide a clear clinical interpretation, the “responder rate” was defined as the percentage of patients who achieved a GAIS score of 3, 4, or 5, rather than the total number of participants who submitted a GAIS evaluation. This specific threshold was used to distinguish participants who showed any degree of clinical improvement from those who remained unaltered or worsened.

#### Morphometric Assessment (3D Imaging)

Three-dimensional facial scans were acquired using the LifeViz^®^ Mini 3D camera system (QuantifiCare, Biot, France). To evaluate longitudinal structural changes, the baseline and follow-up 3D scans were digitally registered and superimposed using the silhouette overlay function in the QuantifiCare 3D analysis software (3D Track module). This alignment was rendered in Clay Mode to eliminate confounding surface texture, skin color, and ambient lighting artifacts, thereby explicitly isolating contour discrepancies over time. Utilizing this standardized 3D framework, the nasofrontal angle and chin volumes were quantified. The nasofrontal angle was measured on the mid-sagittal plane as the angle between the glabella–nasion and nasion–tip lines. For chin volume measurement, 6 regions, including mental crease, mental prominence, apex of chin, submental area, and right and left prejaw areas, were selected based on soft tissue anatomical reference points. Data were normalized by calculating the change ratio (volume/angle change per injected volume) for comparative assessment. Standardized photography, including close-up views of the treated areas and frontal full-face views, was also performed at baseline, Week 4, and Week 24.

#### FACE-Q Satisfaction Questionnaires

Patient-reported satisfaction with both procedure and aesthetic appearance was measured using a 4-point response option set: (1) for Satisfaction with the procedure “very dissatisfied” (Point 1), “somewhat dissatisfied” (Point 2), “somewhat satisfied” (Point 3), and “very satisfied” (Point 4), and (2) for satisfaction with aesthetic appearance “definitely disagree” (Point 1), “somewhat disagree” (Point 2), “somewhat agree” (Point 3), and “definitely agree” (Point 4), and converted to Rasch-transformed scores (0-100).^9^

#### Safety and Pain

AEs and long-term reactions were monitored for 18 months. Procedural pain was assessed immediately postinjection using a 10-point visual analog scale (VAS) ranging from 0 (no pain) to 10 (worst pain).

### Statistical Methods

Statistical analyses were performed using SAS software (version 9.4; SAS Institute Inc., Cary, NC). Continuous variables were summarized descriptively, and pre- to postinjection changes were analyzed using paired *t*-tests or Wilcoxon signed-rank tests, with a 2-sided significance level of .05. Categorical variables were summarized as frequencies and percentages with 95% CIs.

Analyses were conducted at the patient level. For patients treated at both the nose and the chin, an index site was prespecified as the site with the lower baseline FACE-Q satisfaction score to minimize within-patient correlation and ensure independence. Site-specific results were presented as supportive analyses.

Conservative sensitivity analyses were performed for missing data: GAIS missing observations were imputed as nonresponders, and Baseline Observation Carried Forward and Last Observation Carried Forward methods were applied to 3D morphometric variables and FACE-Q scores, respectively. Exploratory subgroup analyses assessed GAIS outcomes by touchup status.

The sample size was determined using a 1-sample proportion test. Assuming a null 18-month GAIS responder rates of 49% based on previous HA filler studies and an expected rate of 84%, 16 patients were required to achieve 80% power at a 2-sided significance level of .05, including a 10% dropout rate.^10,11^

Additionally, to assess the full distribution of the ordinal data, the percentage of participants in each of the 5 individual GAIS categories was evaluated.

## RESULTS

### Demographics

The study enrolled a total of 16 patients (15 female, 1 male) with mean age of 34.38 ± 5.57 years. Treatment was performed on the nose in 9 patients (56.25%), the chin in 4 patients (25.00%), and both the nose and chin in 3 patients (18.75%). These distributions yielded a cumulative total of 12 nose procedures and 7 chin procedures (Table 1).

**Table 1:** Demographics

	Total (*n* = 16)
Age (years)	
Mean (±SD)	34.38 (±5.57)
Median (Q1, Q3)	32.00 (30.5, 41.0)
Gender, *n* (%)	
Male	1 (6.25)
Female	15 (93.75)
Ethnicity, *n* (%)	
Asian	16 (100.00)
Treatment site, *n* (%)	
Nose	9 (56.25)
Chin	4 (25.00)
Nose and chin	3 (18.75)
Injected volume (mL)	
Nose (*n* = 9)	
Mean (±SD)	0.88 (±0.10)
Chin (*n* = 4)	
Mean (±SD)	1.98 (±0.82)
Nose and chin (*n* = 3)	
Mean (±SD)	2.00 (±0.70)

SD, standard deviation.

### The Volume of Filler Administered

The amount of YYS 720 administered varied by treatment site. For the nose procedure (*n* = 12), a mean total injection volume was 0.85 mL (±0.20 mL). Seven out of the 12 patients (58.3%) received the total amount divided into 2 sessions (initial treatment: 0.70 ± 0.18 mL; retouch: 0.26 ± 0.11 mL). For the chin procedures (*n* = 7), a mean total injection volume was 1.66 mL (±0.74 mL), and 2 of the 7 patients (28.6%) received their total volume across 2 sessions (initial treatment: 1.41 ± 0.52 mL; touchup: 0.85 ± 0.21 mL). Patients who underwent simultaneous nose and chin treatment (*n* = 3) received a mean total injection volume of 2.00 mL (±0.70 mL). The mean retouch volumes were 0.26 ± 0.11 mL for the nose (*n* = 7) and 0.85 ± 0.21 mL for the chin (*n* = 2), representing a small fraction of the initial injection volumes (Supplemental Table 1).

### GAIS Responder Rate

As shown in Table 2, for all treated patients (*n* = 16), the GAIS responder rate was 100.00% (16/16) immediately after injection. Although 1 patient missed the Month 12 visit, the responder rate remained at 100.00% (15/15) among those assessed. At the final 18-month follow-up, the study's primary endpoint, all 16 patients were evaluated, and the rate remained high at 81.25% (13/16; 95% CI, 54.35-95.95). The slight decrease at Month 18 reflects a natural waning of the aesthetic effect rather than any loss to follow-up. The chronological pattern of the GAIS responder rate for the site-specific groups mirrored that of the total cohort. For both the nose-only (*n* = 12) and chin-only (*n* = 7) groups, the responder rate remained at 100.00% up to Month 12. At Month 18, these rates slightly decreased to 83.33% (10/12) and 85.71% (6/7), respectively. To investigate whether the amount of filler used for retouching influenced the durability of efficacy at 18 months, patients were categorized based on whether they received a retouch. A comparison of the 18-month GAIS responder rates between these 2 groups revealed no statistically significant differences (Supplemental Table 2), suggesting that the long-term outcomes of YYS 720 observed in this study were independent of the retouch procedure. Additionally, the detailed distribution of participants across all 5 individual GAIS categories at 18 months postinjection is provided in Supplemental Table 3.

**Table 2:** GAIS Responder Rate at Each Time Point

	After injection (Visit 1)	Weeks 2-4 (Visit 2)	Month 3 (Visit 3)	Month 6 (Visit 4)	Month 12 (Visit 5)	Month 18 (Visit 6)
Overall						
Responder/*n*	16/16	16/16	16/16	16/16	15/15	13/16
Responder rate, %	100.00	100.00	100.00	100.00	100.00	81.25
95% CI^a^	(79.41, 100.00)	(79.41, 100.00)	(79.41, 100.00)	(79.41, 100.00)	(78.20, 100.00)	(54.35, 95.95)
Nose						
Responder/*n*	12/12	12/12	12/12	12/12	11/11	10/12
Responder rate, %	100.00	100.00	100.00	100.00	100.00	83.33
95% CI^a^	(73.54, 100.00)	(73.54, 100.00)	(73.54, 100.00)	(73.54, 100.00)	(71.51, 100.00)	(51.59, 97.91)
Chin						
Responder/*n*	7/7	7/7	7/7	7/7	7/7	6/7
Responder rate, %	100.00	100.00	100.00	100.00	100.00	85.71
95% CI^a^	(59.04, 100.00)	(59.04, 100.00)	(59.04, 100.00)	(59.04, 100.00)	(59.04, 100.00)	(42.13, 99.64)

The discrepancy in sample size at Month 12 is because of incomplete data collection.

^a^95% CI is calculated by Clopper–Pearson's method.

### Objective 3D Measurement Analysis

The efficacy of YYS 720 was evaluated through 3D imaging by measuring absolute morphological changes in the nasofrontal angle and chin volume (Tables 3, 4). To account for variability in injection amounts, absolute 3D measurement data were normalized against the total volume of YYS 720 administered by calculating the change ratio (volume/angle change per injected volume), enabling a more precise assessment of filler efficiency (Supplemental Tables 4, 5).

**Table 3:** Nasofrontal Angle Change from Baseline at Each Time Point

	After injection (V1)	Weeks 2-4 (V2)	Month 3 (V3)	Month 6 (V4)	Month 12 (V5)	Month 18 (V6)
*N*	12	12	12	12	10	12
Mean (±SD)	3.56 (±2.16)	4.48 (±3.05)	4.31 (±3.82)	4.02 (±3.56)	3.99 (±2.81)	1.79 (±3.14)
95% CI	(2.19, 4.93)	(2.54, 6.42)	(1.88, 6.74)	(1.76, 6.28)	(1.98, 6.01)	(−0.20, 3.79)
Median (Q1, Q3)	3.96 (1.63, 5.22)	4.43 (3.28, 6.35)	3.99 (2.25, 8.25)	4.08 (1.59, 7.33)	4.31 (1.56, 5.96)	1.44 (0.18, 3.35)
*P*-value	.0001*	.0004*	.0025*	.0024*	.0015*	.0736

The discrepancy in sample size at Month 12 is because of incomplete data collection.

SD, standard deviation.

^*^Statistically significant results (*P* < .05). Changes from before injection were analyzed by paired *t*-test.

**Table 4:** Chin Volume Change from Baseline at Each Time Point

	After injection (V1)	Weeks 2-4 (V2)	Month 3 (V3)	Month 6 (V4)	Month 12 (V5)	Month 18 (V6)
*n*	7	7	7	5	7	7
Mean (±SD)	2.01 (±1.21)	2.33 (±1.09)	2.13 (±0.97)	2.02 (±1.06)	1.93 (±0.94)	1.73 (±1.11)
95% CI	(0.89, 3.13)	(1.32, 3.34)	(1.24, 3.03)	(0.71, 3.33)	(1.06, 2.79)	(0.71, 2.76)
Median (Q1, Q3)	1.64 (0.70, 2.98)	1.97 (1.61, 3.33)	1.86 (1.51, 3.14)	1.75 (1.60, 3.07)	1.61 (1.43, 2.94)	1.57 (0.66, 2.91)
*P*-value*	.0047*	.0013*	.0011*	.0128*	.0016*	.0061*

The discrepancy in sample size at Month 6 is because of incomplete data collection.

SD, standard deviation.

^*^Statistically significant results (*P* < .05). Changes from before injection were analyzed by paired *t*-test.

#### Nasofrontal Angle Change

The mean preinjection nasofrontal angle was 146.07° ± 4.36°. Following injection, the angle demonstrated a significant increase immediately posttreatment, with the mean change from baseline being 3.56 ± 2.16 (*P* = .0001). This increase significantly persisted until Month 12 (3.99 ± 2.81, *P* = .0015). However, at Month 18, statistical significance was no longer observed (*P* = .0736), although the mean angle change remained positive at 1.79 ± 3.14 (Table 3).

#### Chin Volume Change

As shown in Table 4, the mean chin volume change demonstrated a significant increase immediately postinjection (2.01 ± 1.21, *P* = .0047), and it reached its peak level at Weeks 2 to 4 (2.33 ± 1.09, *P* = .0013). This significant increase persisted thereafter, with a continued statistically significant increase confirmed at the final 18-month follow-up (1.73 ± 1.11, *P* = .0061).

### Photography

**Figure 1.**
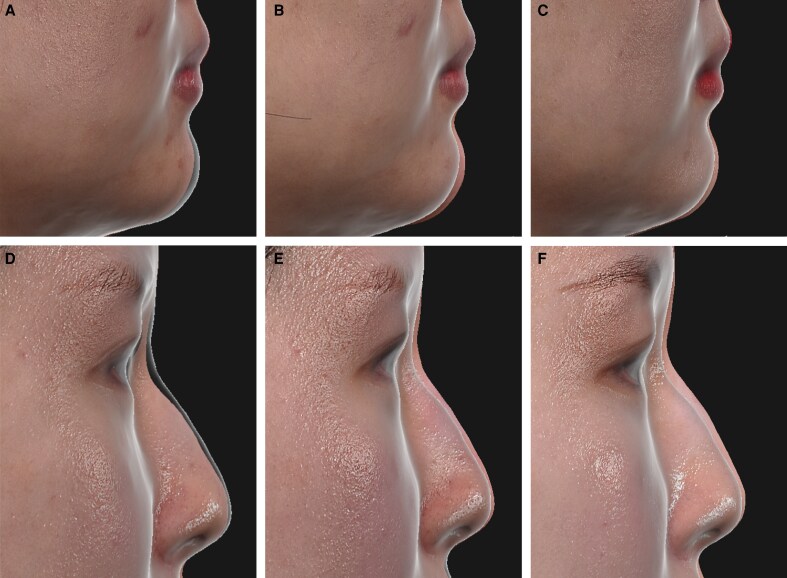
Representative standardized clinical photographs. Patients (A-C, a 24-year-old female; D-F, a 27-year-old female) received (A-C) nose and (D-F) chin augmentation with YYS 720, respectively. Lateral views were captured (A, D) before, (B, E) immediately after the procedure, and (C, F) 18 months posttreatment to illustrate the longitudinal morphological changes. Images were captured using the LifeViz Mini 3D camera system (QuantifiCare, Biot, France). Three-dimensional longitudinal analyses were performed using the QuantifiCare 3D Track software module. Pre- and posttreatment 3-dimensional scans were digitally aligned and superimposed using the silhouette overlay function in Clay Mode to minimize surface texture artifacts and objectively visualize structural and volumetric changes over time.

**Figure 2.**
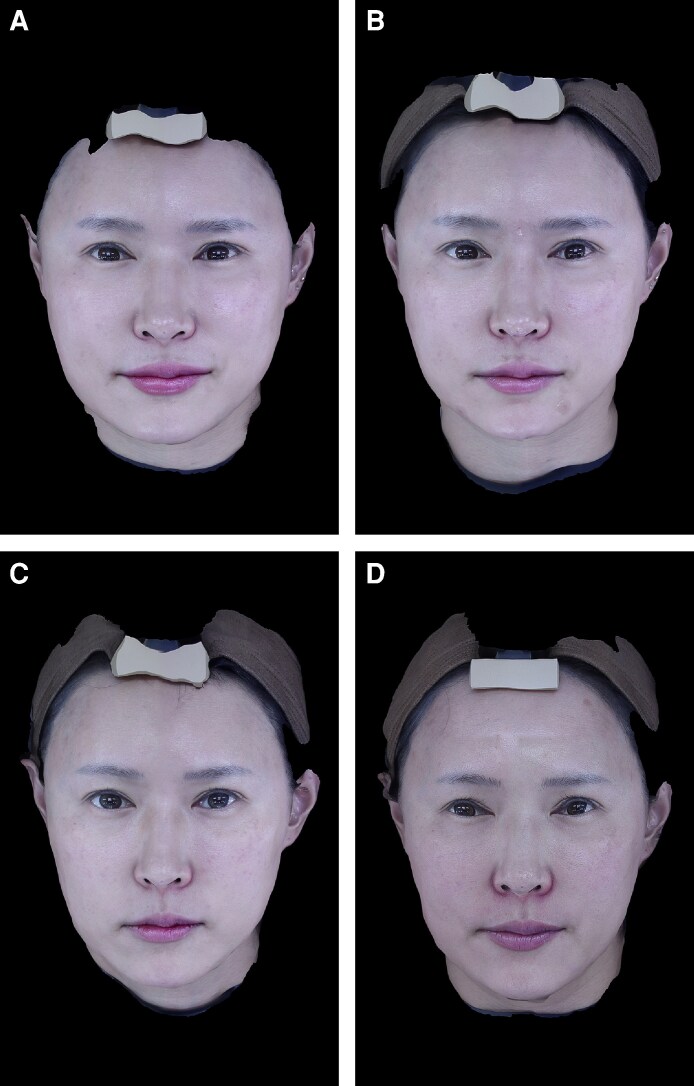
Longitudinal efficacy of YYS 720 in lower face and profile: changes in nose and chin morphometry. A 34-year-old female received both nose and chin augmentations with YYS 720, and representative whole-face photographs were taken at (A) baseline, (B) immediately after the procedure, and (C) 12 and (D) 18 months posttreatment. The localized augmentations demonstrated visual stability and maintenance of the contouring effects throughout the 18-month follow-up period.

Representative lateral views of nose and chin which were captured at baseline and immediately after the procedure and at 18 months posttreatment (Figure 1) demonstrate visible and sustained aesthetic improvements in both the nasofrontal angle and chin projection. Notably, these longitudinal visual enhancements are highly consistent not only with the quantitative 3D image analysis but also with the positive subjective evaluation results from both the investigators and patients, confirming the comprehensive efficacy of YYS 720. Furthermore, full-face images (Figure 2) demonstrate that the localized augmentations remained stable and visually integrated over the 18-month follow-up period.

### Patient-Reported Satisfaction of the Procedures (FACE-Q)

The FACE-Q scales, which were completed by the patients and Rasch-transformed, were measured to assess satisfaction with (1) the procedure including treatment decision and outcome after the procedure, and (2) the aesthetic appearance of the nose and chin areas before and after the procedure. Out of the total 16 enrolled patients, all completed the FACE-Q survey except for 1 participant who had missing data for procedure satisfaction at the Month 12 visit.

#### Satisfaction With the Procedure

The Rasch-transformed score for overall satisfaction with the treatment choice and outcome was substantially high immediately after the procedure, averaging around 87 (decision: 87.44 ± 16.72; outcome: 86.75 ± 16.11), indicating very high procedural contentment. Statistical precision for these satisfaction metrics was confirmed using 95% CIs, which pertain to the distribution of scores among the evaluable cohort at each time point rather than follow-up completion rates. The satisfaction level remained highly stable over time, showing virtually no decline, with the level for each parameter reaching 86.19 ± 21.80 and 85.44 ± 23.64 for decision and outcome even 18 months later, respectively (Supplemental Table 6).

#### Satisfaction With the Aesthetic Appearance

Preprocedure, Rasch-transformed scores for patient satisfaction with the aesthetic appearance of the nose and the chin were 31.42 ± 20.36 and 44.29 ± 12.41, respectively (Supplemental Table 7 and Supplemental Figure 1). Following the filler injection, satisfaction with the aesthetic appearance of both areas significantly increased, but their periods of sustained significance demonstrated contrasting temporal patterns.

A significant increase in postprocedure appearance satisfaction was observed right after the procedure (nose: 83.33 ± 19.81, *P* = .0005; chin: 73.86 ± 23.84, *P* = .0313) up to Month 6 (nose: 86.83 ± 18.58, *P* = .0005; chin 86.00 ± 18.43, *P* = .0156). However, beyond that point, satisfaction varied by treatment site. For nose procedure patients, the FACE-Q score consistently maintained a significant increase through the final 18-month follow-up (80.67 ± 24.35, *P* = .0015). In contrast, for patients with chin procedure, statistical significance was lost after Month 6 (Month 12: 73.29 ± 18.59; Month 18: 74.71 ± 18.04, *P* = .0625 for both time points; Supplemental Table 7).

### Pain Assessment

VAS scores measured immediately postprocedure were confirmed to be very low for patients who underwent both the nose and chin procedures (Table 5). The overall mean VAS score for all patients was 1.69 ± 1.40, indicating similar pain experience between the 2 injection areas. When comparing the mean VAS scores by treatment sites, the scores were 1.92 ± 1.51 for the nose, 1.14 ± 0.69 for the chin, and 1.33 ± 0.58 for the simultaneous nose and chin treatment. In addition, there was no correlation between the administered filler volume and the VAS score (Spearman's *r* = 0.35, *P* = .1798).

**Table 5:** VAS Pain Scale

	Total (*n* = 16)
Nose or chin	
*n*	16
Mean (±SD)	1.69 (±1.40)
Median (Q1, Q3)	1.50 (1, 2)
Nose	
*n*	12
Mean (±SD)	1.92 (±1.51)
Median (Q1, Q3)	2.00 (1, 2)
Chin	
*n*	7
Mean (±SD)	1.14 (±0.69)
Median (Q1, Q3)	1.00 (1, 1.5)
Nose and chin	
*n*	3
Mean (±SD)	1.33 (±0.58)
Median (Q1, Q3)	1.00 (1, 1.5)

SD, standard deviation; VAS, visual analog scale.

### Safety Assessment

Among the 16 patients, postprocedure swelling and tenderness were reported by 3 in the nasal group (*n* = 12) and 1 in the chin group (*n* = 7). These symptoms were resolved within 2 to 7 days for the nose and 3 to 4 days for the chin, with no further complications noted in either group. No long-term AEs, including delayed hypersensitivity reactions or nodules, were observed throughout the 18-month follow-up period.

## DISCUSSION

This study is the first to evaluate the long-term efficacy and safety of YYS-720―a high-viscosity, high-cohesivity HA filler―over 18 months for nose and chin augmentation. Efficacy was systemically assessed through both subjective measures (practitioner-assessed GAIS and patient-assessed FACE-Q) and objective 3D imaging. The results demonstrate the long-term aesthetic efficacy and sustained outcomes of YYS 720. The GAIS responder rate reached 100% through Month 12, and remained high at 84.2% at Month 18. This observed longevity fulfills an important clinical objective for HA fillers in these anatomical regions. Furthermore, the author acknowledges that retouching represents an additional intervention; however, the volumes administered were significantly lower than the initial doses (0.26 ± 0.11 mL for the nose and 0.85 ± 0.21 mL for the chin) and were limited to a final refinement step within 4 weeks postinjection.

The sustained effect of YYS 720 is attributed to its Y-phasic structure, manufactured using the proprietary high-concentration equalized crosslinking method. This process enhances sustainability and cohesivity while limiting the amount of the crosslinker 1,4-butanediol diglycidyl ether, yielding a modification degree (MoD) of 3.3%.^8^ This MoD value contributes to a balanced viscoelasticity and extrusion force, thereby supporting tissue integration. Clinically, the low incidence of procedural pain was demonstrated by a VAS score below 2 points for nasal and chin treatments, which is lower than those reported for HA fillers with similar physicochemical characteristics.^12^ Consequently, these data suggest that YYS 720 provides extended longevity while maintaining a favorable safety profile and low procedural discomfort.

This study utilized 3D imaging analysis to provide an objective, quantitative evaluation of the overall morphological changes. The average nasofrontal angle was 146.07° ± 4.36° at baseline, a value consistent with existing Korean literature (142.5° ± 5.0° to 145.81° ± 6.03°).^13-15^ Following injection, the nasofrontal angle showed a clinically significant increase, reaching a maximum angle of 150.56° (a change of 4.49°) at 2 to 4 weeks, and this improvement was maintained with a mean increase of 1.8° from the baseline even at 18-month follow-up. To the author's knowledge, this represents one of the longer follow-up periods utilizing objective 3D measurements reported for this indication.^16,17^ Concurrently, overall subjective evaluation was affirmed by the FACE-Q results, where Rasch-transformed scores demonstrated the durability of the product's effect: participant satisfaction with the treatment outcome did not diminish over time up to 18 months, maintaining 98.5% of the immediate postprocedure score.

Unlike the overall sustained satisfaction with the procedure, FACE-Q results concerning the contour of the treatment area differed by region. Satisfaction scores for the nose appearance were significantly maintained thoroughly up to 18 months (remaining 2.57 times higher than baseline), although the objective analysis of the nasofrontal angle did not show a statistically significant increase at that time point. Conversely, chin scores were statistically significant only for the initial 6 months, and patients did not report satisfaction at both the 12- and 18-month follow-ups, despite 3D analysis confirming a statistically significant increase in chin volume at these time points.

These contradictory findings can be attributed to several factors. Primarily, the satisfaction with nose appearance (Rasch-transformed score: 31.42) in this study was lower than that for chin appearance (44.29) in the preprocedural FACE-Q survey, and the patient proportion requiring a retouch session was higher for the nose (58.3%, 7 out of 12) compared with the chin (22.2%, 2 out of 9). These observations reflect their higher expectations for the nose, which likely led them to perceive the resulting changes as more substantial aesthetic improvement, thus overcoming the objective loss in nasofrontal angle over time. This tendency aligns with existing reports that nose augmentation procedures are more frequent among Asians than in Western populations.^18^ Additionally, the nasofrontal angle may be insufficient to capture all of the patients’ aesthetic preferences (eg, subtle contour and linear aesthetics), further contributing to the discrepancy between objective measures and subjective satisfaction. For the chin, although volume was objectively maintained as confirmed, the filler may have migrated or spread because of gravity or movement over time, preventing the maintenance of the sharp contour patients desire and thus leading to a decline in their satisfaction. These contrasting clinical and subjective outcomes suggest that to effectively evaluate the long-term efficacy of dermal fillers, it is insufficient to rely solely on objective metrics such as volume or angle. Instead, the success of the aesthetic outcome following the procedure must be assessed multidimensionally, incorporating patient-reported outcomes such as the FACE-Q.

In this study, evaluations were conducted at multiple time points: pretreatment, immediately posttreatment, and at 2 to 4 weeks, 3, 6, 12, and 18 months. Given that this was a pilot study involving only 16 patients, even a small amount of missing data could potentially affect the reliability of the overall findings. To address this and exclude potential bias, supplementary sensitivity analyses on the missing observations were performed. These analyses confirmed that the missing data did not significantly impact on the results for GAIS (Supplemental Table 8), nasofrontal angle (Supplemental Table 9), chin volume (Supplemental Table 10), or FACE-Q scores (Supplemental Table 11), thereby reinforcing the validity of the conclusions.

This study has several limitations that warrant careful consideration when interpreting the findings. First, a significant methodological constraint lies in the potential for observer and attrition biases, which are inherently linked to the small-scale nature of this pilot study and the absence of independent, blinded evaluators. Such biases may influence the subjective assessments of clinical improvement, necessitating a cautious approach to the results. Second, as the research was conducted at a single center with a cohort predominantly composed of female Korean patients, the findings may not be fully generalizable to broader populations with different ethnic backgrounds, skin structures, or aesthetic requirements. Furthermore, because this study evaluated the performance of YYS 720 in isolation, it does not provide a comparative analysis against other established HA filler products, thereby limiting the ability to determine its comparative merit or distinct clinical advantages in a competitive context. Another notable constraint involves the interpretation of long-term responder rates; although the 18-month safety profile was confirmed to be favorable, the unavoidable loss to follow-up over this extended period may introduce variability into the longitudinal efficacy data. Additionally, although the overall retouch rate approached statistical significance (Supplemental Table 2; *P* = .0625), it did not achieve full statistical significance. This outcome should be interpreted with caution, because the study may have been underpowered to detect a true statistical difference because of the fact that only a small subset of patients underwent retouch procedures. In light of these constraints, although this study provides foundational evidence for the use of YYS 720 in facial contouring, larger multicenter trials with more diverse demographics and comparative arms are required to further validate and extend these initial observations.

## CONCLUSIONS

In this pilot study, YYS 720 demonstrated prolonged aesthetic effects and a favorable safety profile at 18 months for both nasal and chin augmentation.

## Supplementary Material

ojag146_Supplementary_Data
