# Supplementary material for: An 18-Month Prospective Evaluation of a Novel Hyaluronic Acid Filler (YYS 720) for 3-Dimensional Nasal and Chin Augmentation
Source: Aesthet Surg J Open Forum. 2026 Jul 14;8:ojag146. doi: 10.1093/asjof/ojag146 (PMC13426315; doi:10.1093/asjof/ojag146)
Supplement: ojag146_Supplementary_Data [file ojag146_supplementary_data.zip › Supplementary Figure Legends.docx]

**Figure Legend for Supplementary Figure S1**

**Supplementary Figure S1.** Rasch-transformed scores for patient satisfaction with the aesthetic appearance of (A) the nose and (B) the chin. The FACE-Q scores were measured by the subjects at pre-procedure (Before injection), and immediately after, and 12 and 18 months after the procedure, and Rasch-transformed. **p* < 0.05, compared to before injection.
