# Supplementary material for: An 18-Month Prospective Evaluation of a Novel Hyaluronic Acid Filler (YYS 720) for 3-Dimensional Nasal and Chin Augmentation
Source: Aesthet Surg J Open Forum. 2026 Jul 14;8:ojag146. doi: 10.1093/asjof/ojag146 (PMC13426315; doi:10.1093/asjof/ojag146)
Supplement: ojag146_Supplementary_Data [file ojag146_supplementary_data.zip › Supplementary Table S1.docx]

Supplementary Table S1. Injected Filler Volume

|  | **Initial Treatment** | **Retouch** | **Total** |
| --- | --- | --- | --- |
| **Nose (mL)** |  |  |  |
| n | 12 | 7 | 12 |
| Mean (± SD) | 0.70 (± 0.18) | 0.26 (± 0.11) | 0.85 (± 0.20) |
| Median (Q1, Q3) | 0.70 (0.60, 0.83) | 0.20 (0.20, 0.35) | 0.90 (0.80, 1.00) |
|  |  |  |  |
| **Chin (mL)** |  |  |  |
| n | 7 | 2 | 7 |
| Mean (± SD) | 1.41 (± 0.52) | 0.85 (± 0.21) | 1.66 (± 0.74) |
| Median (Q1, Q3) | 1.00 (1.00, 1.95) | 0.85 (0.78, 0.93) | 1.70 (1.00, 1.95) |
|  |  |  |  |
| **Nose and Chin (mL)** |  |  |  |
| n | 3 | 2 | 3 |
| Mean (± SD) | 1.53 (± 0.21) | 0.70 (± 0.42) | 2.00 (± 0.70) |
| Median (Q1, Q3) | 1.60 (1.45, 1.65) | 0.70 (0.55, 0.85) | 2.00 (1.65, 2.35) |
