# Supplementary material for: An 18-Month Prospective Evaluation of a Novel Hyaluronic Acid Filler (YYS 720) for 3-Dimensional Nasal and Chin Augmentation
Source: Aesthet Surg J Open Forum. 2026 Jul 14;8:ojag146. doi: 10.1093/asjof/ojag146 (PMC13426315; doi:10.1093/asjof/ojag146)
Supplement: ojag146_Supplementary_Data [file ojag146_supplementary_data.zip › Supplementary Table S10.docx]

Supplementary Table S10. Sensitivity Analysis of Changes in Chin Volume from Baseline: Applying BOCF for Missing Data

|  | **After injection (V1)** | **Week 2-4 (V2)** | **Month 3 (V3)** | **Month 6 (V4)** | **Month 12 (V5)** | **Month 18 (V6)** |
| --- | --- | --- | --- | --- | --- | --- |
| n | 7 | 7 | 7 | 7 | 7 | 7 |
| Mean (± SD) | 2.01 (±1.21) | 2.33 (±1.09) | 2.13 (±0.97) | 1.93 (±0.94) | 1.93 (±0.94) | 1.73 (±1.11) |
| 95% CI | [0.89, 3.13] | [1.32, 3.34] | [1.24, 3.03] | [1.06, 2.79] | [1.06, 2.79] | [0.71, 2.76] |
| Median (Q1, Q3) | 1.64 (0.70, 2.98) | 1.97 (1.61, 3.33) | 1.86 (1.51, 3.14) | 1.61 (1.43, 2.94) | 1.61 (1.43, 2.94) | 1.57 (0.66, 2.91) |
| p-value* | **0.0047** | **0.0013** | **0.0011** | **0.0268** | **0.0016** | **0.0061** |

**Changes from before injection were analyzed by paired t-test; Statistically significant results are presented in bold (p < 0.05).*

***Abbreviation: BOCF****, Baseline Observation Carried Forward*
