# Supplementary material for: An 18-Month Prospective Evaluation of a Novel Hyaluronic Acid Filler (YYS 720) for 3-Dimensional Nasal and Chin Augmentation
Source: Aesthet Surg J Open Forum. 2026 Jul 14;8:ojag146. doi: 10.1093/asjof/ojag146 (PMC13426315; doi:10.1093/asjof/ojag146)
Supplement: ojag146_Supplementary_Data [file ojag146_supplementary_data.zip › Supplementary Table S2.docx]

Supplementary Table S2. GAIS Responder Rate at 18 months, Stratified by Retouch Subgroup

|  | **Without Retouch** | **With Retouch** |
| --- | --- | --- |
| **Overall** |  |  |
| responder / n | 9 / 9 | 4 / 7 |
| responder rate, % | 100.00 | 57.14 |
| 95% CI* | [66.37, 100.00] | [18.41, 90.10] |
| p-value** |  | 0.0625 |
| **Nose** |  |  |
| responder / n | 5 /5 | 5 / 7 |
| responder rate, % | 100.00 | 71.43 |
| 95% CI* | [47.82, 100.00] | [29.04, 96.33] |
| p-value** |  | 0.4697 |
| **Chin** |  |  |
| responder / n | 5 / 5 | 1 / 2 |
| responder rate, % | 100.00 | 50.00 |
| 95% CI* | [47.82, 100.00] | [1.26, 98.74] |
| p-value** |  | 0.2857 |

**95% CI is calculated by Clopper-Pearson’**s method.*

***Differences in GAIS responder rates between the ‘without retouch’ and ‘with retouch’ subgroups were analyzed by Fisher’s exact test.*
