# Supplementary material for: An 18-Month Prospective Evaluation of a Novel Hyaluronic Acid Filler (YYS 720) for 3-Dimensional Nasal and Chin Augmentation
Source: Aesthet Surg J Open Forum. 2026 Jul 14;8:ojag146. doi: 10.1093/asjof/ojag146 (PMC13426315; doi:10.1093/asjof/ojag146)
Supplement: ojag146_Supplementary_Data [file ojag146_supplementary_data.zip › Supplementary Table S3.docx]

**Supplementary Table S3. Distribution of Participants across All Five Individual GAIS* Categories at 18 Months Post-Injection**

|  | | **1**  Worsened | **2**  Unaltered | **3**  Improved | **4**  Very Improved | **5**  Exceptional Improvement |
| --- | --- | --- | --- | --- | --- | --- |
|  |  | |  |  |  |  |
| **Overall (N=16)** | |  |  |  |  |  |
| Participants, n | | 0 | 3 | 9 | 4 | 0 |
| Participants, % | | 0.00 | 18.75 | 56.25 | 25.00 | 0.00 |
| 95% CI** | | [0.00, 20.59] | [4.05, 45.65] | [29.88, 80.25] | [7.27, 52.38] | [0.00, 20.59] |
| **Nose (N=12)** | |  |  |  |  |  |
| Participants, n | | 0 | 2 | 7 | 3 | 0 |
| Participants, % | | 0.00 | 16.67 | 58.33 | 25.00 | 0.00 |
| 95% CI | | [0.00, 26.47] | [2.09, 48.41] | [27.67, 84.83] | [5.49, 57.19] | [0.00, 26.47] |
| **Chin (N=7)** | |  |  |  |  |  |
| Participants, n | | 0 | 1 | 4 | 2 | 0 |
| Participants, % | | 0.00 | 14.29 | 57.14 | 28.57 | 0.00 |
| 95% CI | | [0.00, 40.96] | [0.36, 57.87] | [18.41, 90.10] | [3.67, 70.96] | [0.00, 40.96] |

******GAIS, Global Aesthetic Improvement Scale***

*****CI, confidence interval; 95% CI is calculated using the Clopper–Pearson method****.*
