# Supplementary material for: An 18-Month Prospective Evaluation of a Novel Hyaluronic Acid Filler (YYS 720) for 3-Dimensional Nasal and Chin Augmentation
Source: Aesthet Surg J Open Forum. 2026 Jul 14;8:ojag146. doi: 10.1093/asjof/ojag146 (PMC13426315; doi:10.1093/asjof/ojag146)
Supplement: ojag146_Supplementary_Data [file ojag146_supplementary_data.zip › Supplementary Table S4.docx]

Supplementary Table S4. Ratio of Nasofrontal Angle Change from Before Injection Relative to Injection Volume* at Each Timepoint

|  | **After injection (V1)** | **Week 2-4 (V2)** | **Month 3 (V3)** | **Month 6 (V4)** | **Month 12 (V5)** | **Month 18 (V6)** |
| --- | --- | --- | --- | --- | --- | --- |
| n | 12 | 12 | 12 | 12 | 10 | 12 |
| Mean (± SD) | 5.45 (± 3.28) | 5.22 (± 3.71) | 5.00 (± 4.77) | 4.58 (± 4.61) | 4.55 (± 4.24) | 1.36 (± 5.38) |
| 95% CI | [3.37, 7.54] | [2.86, 7.57] | [1.97, 8.04] | [1.65, 7.51] | [1.52, 7.58] | [-2.05, 4.78] |
| Median (Q1, Q3) | 5.66 (3.26, 7.50) | 5.02 (3.28, 7.63) | 4.28 (2.30, 9.71) | 4.22 (1.64, 8.58) | 4.56 (1.56, 6.62) | 1.53 (0.21, 3.84) |
| p-value** | **0.0001** | **0.0005** | **0.0040** | **0.0055** | **0.0079** | 0.0771^#^ |

**Ratio = nasofrontal angle change / injection volume*

***Changes from before injection were analyzed by paired t-test or Wilcoxon signed-rank test (# for Wilcoxon signed-rank test); Statistically significant results are presented in bold (p < 0.05).*

*The discrepancy in sample size at Month 12 is due to incomplete data collection.*
