# Supplementary material for: An 18-Month Prospective Evaluation of a Novel Hyaluronic Acid Filler (YYS 720) for 3-Dimensional Nasal and Chin Augmentation
Source: Aesthet Surg J Open Forum. 2026 Jul 14;8:ojag146. doi: 10.1093/asjof/ojag146 (PMC13426315; doi:10.1093/asjof/ojag146)
Supplement: ojag146_Supplementary_Data [file ojag146_supplementary_data.zip › Supplementary Table S5.docx]

Supplementary Table S5. Ratio of Chin Volume Change from Before Injection Relative to Injection Volume* at Each Timepoint

|  | **After injection (V1)** | **Week 2-4 (V2)** | **Month 3 (V3)** | **Month 6 (V4)** | **Month 12 (V5)** | **Month 18 (V6)** |
| --- | --- | --- | --- | --- | --- | --- |
| n | 7 | 7 | 7 | 5 | 7 | 7 |
| Mean (± SD) | 1.43 (± 0.82) | 1.52 (± 0.85) | 1.41 (± 0.83) | 1.47 (± 0.97) | 1.28 (± 0.83) | 1.19 (± 0.93) |
| 95% CI | [0.67, 2.18] | [0.73, 2.31] | [0.64, 2.17] | [0.27, 2.67] | [0.51, 2.04] | [0.33, 2.05] |
| Median (Q1, Q3) | 1.49 (0.70, 1.87) | 1.16 (0.87, 1.84) | 1.09 (0.79, 1.59) | 1.03 (1.02, 1.60) | 0.98 (0.75, 1.61) | 0.71 (0.42, 1.70) |
| p-value** | **0.0037** | **0.0033** | **0.0156^#^** | **0.0274** | **0.0065** | **0.0149** |

**Ratio = chin volume change / injection volume*

***Changes from before injection were analyzed by paired t-test or Wilcoxon signed-rank test (# for Wilcoxon signed-rank test); Statistically significant results are presented in bold (p < 0.05).*

*The discrepancy in sample size at Month 6 is due to incomplete data collection.*
