# Supplementary material for: An 18-Month Prospective Evaluation of a Novel Hyaluronic Acid Filler (YYS 720) for 3-Dimensional Nasal and Chin Augmentation
Source: Aesthet Surg J Open Forum. 2026 Jul 14;8:ojag146. doi: 10.1093/asjof/ojag146 (PMC13426315; doi:10.1093/asjof/ojag146)
Supplement: ojag146_Supplementary_Data [file ojag146_supplementary_data.zip › Supplementary Table S6.docx]

Supplementary Table S6. Changes in Rasch-Transformed FACE-Q Scores for Overall Treatment at Each Timepoint

|  | **After injection (V1)** | **Week 2-4 (V2)** | **Month 3 (V3)** | **Month 6 (V4)** | **Month 12 (V5)** | **Month 18 (V6)** |
| --- | --- | --- | --- | --- | --- | --- |
| **Satisfaction with Decision** | | | | | | |
| n | 16 | 16 | 16 | 16 | 15 | 16 |
| Mean (± SD) | 87.44 (± 16.72) | 86.69 (± 18.48) | 84.63 (± 19.38) | 87.81 (± 18.72) | 80.27 (± 20.68) | 86.19 (± 21.80) |
| 95% CI | [78.53, 96.35] | [76.84, 96.54] | [74.30, 94.95] | [77.84, 97.79] | [68.82, 91.72] | [74.57, 97.81] |
| Median (Q1, Q3) | 100 (75, 100) | 100 (64, 100) | 100 (61.5, 100) | 100 (64, 100) | 90 (59, 100) | 100 (61.5, 100) |
|  |  |  |  |  |  |  |
| **Satisfaction with Outcome** | | | | | | |
| n | 16 | 16 | 16 | 16 | 15 | 16 |
| Mean (± SD) | 86.75 (± 16.11) | 82.00 (± 18.23) | 79.81 (± 19.63) | 85.88 (± 19.31) | 78.33 (± 22.39) | 85.44 (± 23.64) |
| 95% CI | [78.17, 95.33] | [72.29, 91.71] | [69.35, 90.27] | [75.58, 96.17] | [65.94, 90.73] | [72.84, 98.04] |
| Median (Q1, Q3) | 100 (73, 100) | 83 (61, 100) | 79 (59, 100) | 100 (65.5, 100) | 79 (59, 100) | 100 (66, 100) |

*The discrepancy in sample size at Month 12 is due to incomplete data collection.*
