# Supplementary material for: An 18-Month Prospective Evaluation of a Novel Hyaluronic Acid Filler (YYS 720) for 3-Dimensional Nasal and Chin Augmentation
Source: Aesthet Surg J Open Forum. 2026 Jul 14;8:ojag146. doi: 10.1093/asjof/ojag146 (PMC13426315; doi:10.1093/asjof/ojag146)
Supplement: ojag146_Supplementary_Data [file ojag146_supplementary_data.zip › Supplementary Table S7.docx]

Supplementary Table S7. Changes in Rasch-Transformed FACE-Q Scores by Injection Site at Each Timepoint

|  | **Before injection** | **After injection (Visit 1)** | **Week 2-4**  **(Visit 2)** | **Month 3**  **(Visit 3)** | **Month 6**  **(Visit 4)** | **Month 12 (Visit 5)** | **Month 18**  **(Visit 6)** |
| --- | --- | --- | --- | --- | --- | --- | --- |
| **Satisfaction with Nose** | | | | | | | |
| n | 12 | 12 | 12 | 12 | 12 | 12 | 12 |
| Mean (± SD) | 31.42 (± 20.36) | 83.33 (± 19.81) | 88.50 (± 19.01) | 83.00 (± 19.92) | 86.83 (± 18.58) | 82.75 (± 24.25) | 80.67 (± 24.35) |
| 95% CI | [18.48, 44.35] | [70.75, 95.92] | [76.42, 100.58] | [70.34, 95.66] | [75.03, 98.64] | [67.35, 98.15] | [65.19, 96.14] |
| Median (Q1, Q3) | 37 (15, 44.5) | 95 (59, 100) | 100 (74, 100) | 95 (58, 100) | 100 (68, 100) | 100 (58, 100) | 100 (58, 100) |
| p-value* |  | **0.0005** | **0.0005** | **0.0005** | **0.0005** | **0.0015** | **0.0015** |
|  |  |  |  |  |  |  |  |
| **Satisfaction with Chin** | | | | | | | |
| n | 7 | 7 | 7 | 7 | 7 | 7 | 7 |
| Mean (± SD) | 44.29 (± 12.41) | 73.86 (± 23.84) | 78.14 (± 20.59) | 79.00 (± 19.92) | 86.00 (± 18.43) | 73.29 (± 18.59) | 74.71 (± 18.04) |
| 95% CI | [32.81, 55.76] | [51.81, 95.91] | [59.10, 97.19] | [60.57, 97.43] | [68.96, 103.04] | [56.09, 90.48] | [58.03, 91.40] |
| Median (Q1, Q3) | 37 (33, 60) | 79 (60, 100) | 67 (60, 100) | 70 (60, 100) | 100 (63, 100) | 63 (60, 100) | 70 (60, 100) |
| p-value* |  | **0.0313** | **0.0156** | **0.0156** | **0.0156** | 0.0625 | 0.0625 |

**Changes from before injection were analyzed by Wilcoxon signed-rank test; Statistically significant results are presented in bold (p < 0.05).*
