# Supplementary material for: An 18-Month Prospective Evaluation of a Novel Hyaluronic Acid Filler (YYS 720) for 3-Dimensional Nasal and Chin Augmentation
Source: Aesthet Surg J Open Forum. 2026 Jul 14;8:ojag146. doi: 10.1093/asjof/ojag146 (PMC13426315; doi:10.1093/asjof/ojag146)
Supplement: ojag146_Supplementary_Data [file ojag146_supplementary_data.zip › Supplementary Table S8.docx]

Supplementary Table S8. Sensitivity Analysis of GAIS Responder Rates: Considering Missing Observations as Non-responders

|  | **After injection**  **(Visit 1)** | **Week 2-4**  **(Visit 2)** | **Month 3**  **(Visit 3)** | **Month 6**  **(Visit 4)** | **Month 12**  **(Visit 5)** | **Month 18**  **(Visit 6)** |
| --- | --- | --- | --- | --- | --- | --- |
|  |  |  |  |  |  |  |
| **Overall** |  |  |  |  |  |  |
| responder / n | 16 / 16 | 16 / 16 | 16 / 16 | 16 / 16 | 15 / 16 | 13 / 16 |
| responder rate, % | 100.00 | 100.00 | 100.00 | 100.00 | 93.75 | 81.25 |
| 95% CI* | [79.41, 100.00] | [79.41, 100.00] | [79.41, 100.00] | [79.41, 100.00] | [69.77, 99.84] | [54.35, 95.95] |
| **Nose** |  |  |  |  |  |  |
| responder / n | 12 / 12 | 12 / 12 | 12 / 12 | 12 / 12 | 11 / 12 | 10 / 12 |
| responder rate, % | 100.00 | 100.00 | 100.00 | 100.00 | 91.67 | 83.33 |
| 95% CI* | [73.54, 100.00] | [73.54, 100.00] | [73.54, 100.00] | [73.54, 100.00] | [61.52, 99.79] | [51.59, 97.91] |
| **Chin** |  |  |  |  |  |  |
| responder / n | 7 / 7 | 7 / 7 | 7 / 7 | 7 / 7 | 7 / 7 | 6 / 7 |
| responder rate, % | 100.00 | 100.00 | 100.00 | 100.00 | 100.00 | 85.71 |
| 95% CI* | [59.04, 100.00] | [59.04, 100.00] | [59.04, 100.00] | [59.04, 100.00] | [59.04, 100.00] | [42.13, 99.64] |

**95% CI is calculated by Clopper-Pearson’**s method.*
