# Supplementary material for: An 18-Month Prospective Evaluation of a Novel Hyaluronic Acid Filler (YYS 720) for 3-Dimensional Nasal and Chin Augmentation
Source: Aesthet Surg J Open Forum. 2026 Jul 14;8:ojag146. doi: 10.1093/asjof/ojag146 (PMC13426315; doi:10.1093/asjof/ojag146)
Supplement: ojag146_Supplementary_Data [file ojag146_supplementary_data.zip › Supplementary Table S9.docx]

Supplementary Table S9. Sensitivity Analysis of Changes in Nasofrontal Angle from Baseline: Applying BOCF for Missing Data

|  | **After injection (V1)** | **Week 2-4 (V2)** | **Month 3 (V3)** | **Month 6 (V4)** | **Month 12 (V5)** | **Month 18 (V6)** |
| --- | --- | --- | --- | --- | --- | --- |
| n | 12 | 12 | 12 | 12 | 12 | 12 |
| Mean (± SD) | 3.56 (±2.16) | 4.48 (±3.05) | 4.31 (±3.82) | 4.02 (±3.56) | 3.33 (±2.98) | 1.79 (±3.14) |
| 95% CI | [2.19, 4.93] | [2.54, 6.42] | [1.88, 6.74] | [1.76, 6.28] | [1.43, 5.22] | [-0.20, 3.79] |
| Median (Q1, Q3) | 3.96 (1.63, 5.22) | 4.43 (3.28, 6.35) | 3.99 (2.25, 8.25) | 4.08 (1.59, 7.33) | 3.70 (0.75, 5.33) | 1.44 (0.18, 3.35) |
| p-value* | **0.0001** | **0.0004** | **0.0025** | **0.0024** | **0.0026** | 0.0736 |

**Changes from before injection were analyzed by paired t-test; Statistically significant results are presented in bold (p < 0.05).*

***Abbreviation: BOCF****, Baseline Observation Carried Forward*
