# Supplementary figures and images for: An 18-Month Prospective Evaluation of a Novel Hyaluronic Acid Filler (YYS 720) for 3-Dimensional Nasal and Chin Augmentation
Source: Aesthet Surg J Open Forum. 2026 Jul 14;8:ojag146. doi: 10.1093/asjof/ojag146 (PMC13426315; doi:10.1093/asjof/ojag146)

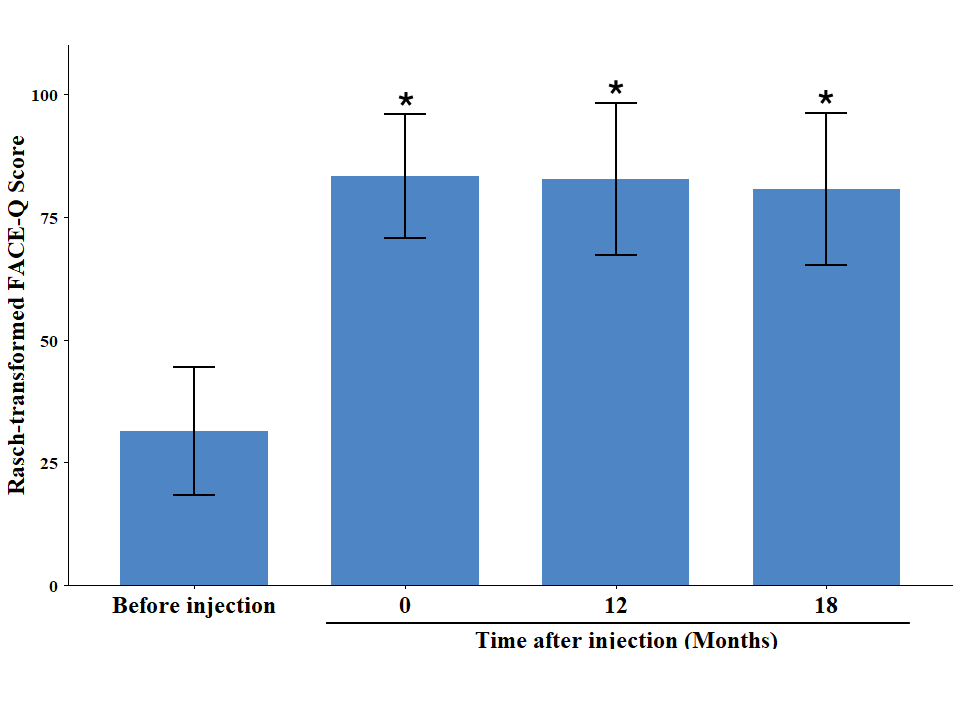

Supplement: ojag146_Supplementary_Data [file ojag146_supplementary_data.zip › Supplementary Figure S1_A.tiff]

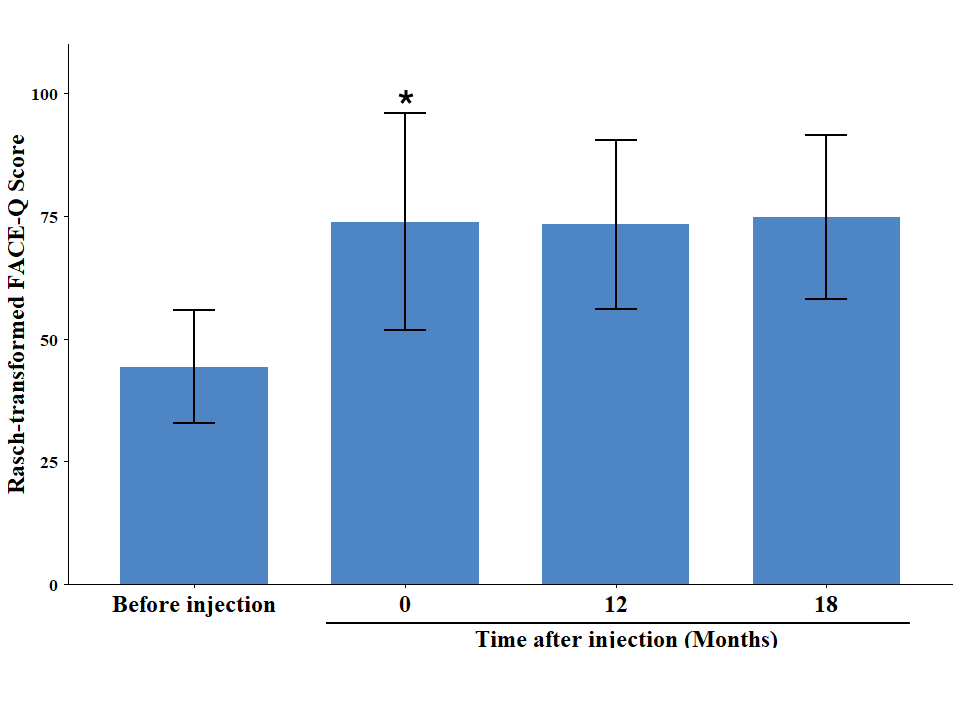

Supplement: ojag146_Supplementary_Data [file ojag146_supplementary_data.zip › Supplementary Figure S1_B.tiff]
